# Pain catastrophising, body mass index and depressive symptoms are associated with pain severity in tertiary referral orthopaedic foot/ankle patients

**DOI:** 10.1186/s13047-022-00536-5

**Published:** 2022-05-06

**Authors:** Matthew Holt, Caitlin L. Swalwell, Gayle H. Silveira, Vivienne Tippett, Tom P. Walsh, Simon R. Platt

**Affiliations:** 1 Department of Orthopaedics, Gold Coast University Hospital, Southport, Queensland 4215 Australia; 2 Griffith University, School of Medicine, Southport, Queensland 4215 Australia; 3 Queensland University of Technology (QUT), Faculty of Health, School of Clinical Sciences, Kelvin Grove, Queensland 4059 Australia; 4 Department of Orthopaedics & Trauma, Northern Adelaide Local Health Network, South Australia, 5112 Australia

**Keywords:** Foot, Ankle, Mental health, Depression, Body mass index, Musculoskeletal pain

## Abstract

**Introduction:**

Patients with chronic foot/ankle pain are often referred for orthopaedic assessment. Psychological vulnerabilities influence pain states (including foot and ankle), therefore this study aimed to establish the prevalence and relative importance of compromised psychological health to perceived foot/ankle pain severity in people referred to an orthopaedic foot and ankle clinic with non-urgent presentations.

**Methods:**

Patients with triaged non-urgent foot/ankle referrals to the Department of Orthopaedics at Gold Coast University Hospital were recruited over a 12-month period and completed the *Manchester-Oxford Foot and Ankle Questionnaire* which was the primary measure. Participants also completed questionnaires assessing their anthropometric, demographic and health characteristics (*Self-Administered Comorbidity Questionnaire*) as well as measures of health-related quality of life (*EuroQol-5-Dimensions-5-Level Questionnaire* and *EQ Visual Analogue Scale*) and psychological health (*Center for Epidemiological Studies-Depression scale*, *Pain Catastrophizing Scale* and *Central Sensitization Inventory*)*.* Descriptive statistics were used to summarise participant characteristics and a hierarchical multiple linear regression was employed to establish the extent to which psychological variables explain additional variance in foot/ankle pain severity beyond the effects of participant characteristics (age, sex, body mass index (BMI)).

**Results:**

One hundred and seventy-two adults were recruited ((64.0% female), median (IQR) age 60.9 (17.7) years and BMI 27.6 (7.5) kg/m^2^). Specific psychological comorbidities were prevalent including depressive symptoms (48%), central sensitisation (38%) and pain catastrophising (24%). Age, sex and BMI accounted for 11.7% of the variance in MOXFQ-index and psychological variables accounted for an additional 28.2%. Pain catastrophising was the most significant independent predictor of foot/ankle pain severity (accounting for 14.4% of variance), followed by BMI (10.7%) and depressive symptoms (2.3%).

**Conclusions:**

This study demonstrated that specific psychological comorbidities and increased BMI are common in this cohort and that these factors are associated with the symptoms for which patients are seeking orthopaedic assessment. This knowledge should prompt clinicians to routinely consider the psychosocial components of patient presentations and develop non-operative and pre-operative treatment strategies which consider these factors with the goal of improving overall patient outcomes.

## Introduction

Foot pain affects one in four, and ankle pain one in six, adults aged 45 years and over [1]. Both are associated with high burden of disease [2, 3]. Foot/ankle pain has been associated with fat and body mass index [4], increased age [5], female gender [5] and a range of psychosocial factors including depression and anxiety [6] as well as reduced health-related quality of life (HRQoL) [5]. In Australia, many people with foot/ankle pain will have initial assessment and treatment in community primary care. A substantial proportion will however be referred to specialist orthopaedic clinics for assessment. They are triaged based on clinical urgency with those considered non-urgent, despite often having chronic pain and/or some level of disability, often subjected to long waiting periods. It has been shown, that amongst patients triaged as non-urgent the proportion who are ultimately treated operatively is as low as 20% [7, 8]. Improving understanding of the clinical characteristics of this non-urgent group, including any non-surgical factors contributing to their pain, is important as it may offer the opportunity to institute early and targeted treatment strategies while they await an orthopaedic surgical assessment.

The reason for low surgical conversion rates in this group is likely multifactorial and may be in part related to the surgeons’ reluctance to operate on patients with discordance between orthopaedic pathology and pain severity. Patients referred to specialist clinics may believe that their pain is primarily generated mechanically in their foot/ankle and evidence suggests that patients tend to have higher expectations of the benefits of surgery than do surgeons [9]. However, research in recent decades has considerably improved clinicians’ understanding of pain as an output of the central nervous system, subject to modification by local and central mediators. Chronic musculoskeletal pain (including foot and ankle pain) is the product of a complex and reciprocal interplay between physiological factors and psychosocial factors including depression and anxiety, poor sleep, catastrophising, personality vulnerabilities, social isolation and occupational and/or socio-economic risk factors [9–15].

In many cases, the psychosocial elements of a patient’s pain presentation can be prognostic in the experience of pain and can negatively influence the likelihood of functional recovery [16, 17]. Clinically diagnosed major depression has a well-established relationship with chronic pain [18] and evidence suggests that depressive symptoms are a possible comorbidity for patients with foot/ankle conditions. For example, one recent study suggested that pre-operative depressive symptoms are associated with worse post-operative pain following total ankle arthroplasty [19] and patients with end-stage ankle arthrosis demonstrate significantly worse mental component summary scores in the 36-Item Short Form Survey Instrument (SF-36) than those with end-stage hip arthrosis, despite showing similar SF-36 physical component scores [2]. Treatment of comorbid depression has also been associated with improved clinical and social/occupational outcomes in a general chronic pain cohort [20]. However the prevalence and modifiability of depression in patients with foot/ankle pain is not yet well understood.

Other psychological features such as pain catastrophising and sensitisation have now also been linked to musculoskeletal pain and surgical outcomes [21]. In knee arthroplasty, for example, there is evidence that pain catastrophising may stand out amongst several possible psychological vulnerabilities as being associated with worse pain and physical function pre- and post-operatively [22] and predictive of poor surgical outcomes [23]. Catastrophising has also been previously associated with increased foot pain [24] and worse patient-reported outcomes (on the Foot and Ankle Outcome Score) including pain following foot/ankle surgery [25]. Pain catastrophising represents a negative mental schema which research suggests is modifiable [26, 27]. Measuring catastrophising, if it leads to interventions aimed at modifying catastrophising in patients pre-operatively, may therefore have potential to inform strategies for positively influencing post-operative outcomes.

This study aims to; (a) undertake an exploratory investigation of the prevalence of psychological health features that may be contributing to foot and ankle pain, and (b) to delineate the contribution and relative importance of these factors to foot and ankle pain presentations.

## Methods

### Study population

Patients referred to a tertiary referral hospitals’ Department of Orthopaedics at Gold Coast University Hospital from May 2019 through April 2020 were invited to participate in this cross-sectional study. Upon receipt of referral, the foot and ankle orthopaedic consultants triage patients as Category 1 (urgent), Category 2 (semi-urgent) and Category 3 (non-urgent) which is the standard of practice for specialist hospital referrals in the Australian public health system. Category 1 and 2 is applied to patients triaged as having a condition which may deteriorate if not reviewed in an urgent (i.e. next available or within 30 days) or semi urgent (i.e. within 90 days) manner, respectively. Category 3 is applied to referrals for conditions considered not life or limb threatening and unlikely to deteriorate or have increased morbidity for which review in a specialist clinic/service is recommended within 365 days of being added to the waiting-list.

### Participant recruitment

All patients triaged to Category 3 during the study period were posted a letter of invitation to participate in the study, participant information sheet and each of the study questionnaires to complete at a single time point. The information sheet clearly outlined that return mailing of their responses in the stamped envelope provided within would constitute consent to participate in the study unless they specified otherwise. The responses were manually entered into an electronic numeric coding spreadsheet for data analysis. To determine the presence of self-selection bias, the age, sex, and socio-economic disadvantage were compared between the responders and non-responders. Gold Coast Hospital and Health Service Human Research Ethics Committee granted ethical approval (Study ID #45244) for this study.

### Inclusion and exclusion criteria

All adults with Category 3 foot/ankle referrals were included in this study. Patients triaged as Category 1 or 2 by the foot and ankle orthopaedic consultants, or patients unable to provide consent (e.g unable to understand written English) or unwilling to provide consent were excluded.

### Anthropometry, demographics and general health

Participants self-reported their age, sex, height, weight, duration of foot/ankle symptoms, current analgesic medication use (paracetamol, oral non-steroidal anti-inflammatory drugs, codeine/other opioids), and smoking status (current smoker, ex-smoker, non-smoker). Duration of foot/ankle pain was collected in year/months and analgesia use was specific for current foot/ankle pain. BMI was calculated by dividing self-reported weight (kg) by height (m^2^). Socio-economic disadvantage was estimated from each participant’s postcode using the Index of Relative Socio-economic Disadvantage (IRSD) from the Australian Bureau of Statistics [28]. Possible scores range from 1 to 10, where 10 is the least disadvantaged.

Current medical conditions were collected via a modified version of the *Self-Administered Comorbidity Questionnaire* (SACQ) [29]. The SACQ comprises 13 forced-choice items (each pertaining to common medical conditions) and one free response item (allowing patients to self-report additional medical complaints). The presence of comorbidities (defined as one or more medical conditions) was calculated by summing the total responses to all 13 forced-choice items and any free response items.

Overall HRQoL was assessed via the *EuroQol-5-Dimensions-5-Level Questionnaire* (EQ-5D-5L) and EQ Visual Analogue Scale (EQ-VAS). The EQ-5D-5L comprises five dimensions (mobility, self-care, usual activities, pain/discomfort and anxiety/depression). Participants indicated their health state across each dimension by ticking the most appropriate statement (from no problems to extreme problems). The EQ-VAS asks participants to self-report their HRQoL using a vertical VAS with endpoints labelled ‘best imaginable health state’ (100) and ‘worst imaginable health state’ (0); thus, the EQ-VAS can be used as a quantitative measure (0–100) of a participant’s perceived health status.

### Pain and disability

The MOXFQ was used to assess the region-specific impact of foot/ankle symptoms on pain, function and social interaction. This questionnaire comprises 16-items across three separate domains, walking/standing problems (seven items), foot/ankle pain (five items) and social interaction (four items); and has been found to be a reliable and valid assessment of foot/ankle pain severity [30]. The MOXFQ-index (an overall summary score ranging from 0 to 100, where 100 is the most severe) was used as the primary measure for all foot/ankle pain severity analyses. Individual domain scores have also been used to report level/type of disability. For participants with bilateral foot/ankle pain, MOXFQ questionnaires were completed twice (one for each limb), and to avoid violating the assumption of independence, only data for the more painful limb were used in the regression analysis. To determine the prevalence of bilateral foot pain in our sample, a MOXFQ-index score of 20 or above for each limb was considered to constitute clinically significant foot pain.

Participants were asked to identify the presence of pain at other joints (both hands, elbows, shoulders, hips, knees, along with the cervical and lumbar spine) that they have had on most days of the week during the past one-month. The total number of joints affected were summed (range 0–12). Multi-site pain was defined as pain at four or more joints.

### Psychological health (depressive symptoms, pain catastrophising, central sensitisation syndrome)

Depressive symptom presence was assessed using the 20-item *Center for Epidemiological Studies-Depression* (CES-D) scale. Participants were asked to indicate how often they have experienced symptoms associated with depression (e.g., loneliness) over the past week. Items are scored using a 4-point Likert scale anchored 0 (rarely or none of the time / less than 1 day) to 3 (all of the time / 5–7 days). Total possible scores range from 0 to 60; and scores ≥16 were considered indicative of depressive symptom presence [31].

The influence of catastrophising on perceptions of painful experiences was established via the 13-item *Pain Catastrophizing Scale* (PCS). This questionnaire has participants reflect on prior painful experiences and indicate the extent to which they have experienced each of 13 thoughts/feelings during these episodes of pain. Responses are scored using 5-point Likert scale anchored 0 (not at all) to 4 (all the time) and items 8 and 12 were rescored as per a previous recommendation [32]. This yielded an overall catastrophising score (0–52), and three subscale scores: Rumination (0–16), magnification (0–12), and helplessness (0–24). In line with existing recommendations [33], a participant scoring ≥30 with the PCS was classified as exhibiting pain catastrophisation.

The two-part *Central Sensitization Inventory* (CSI) was used to establish the role of central mediation on participant pain. Only Part A was used in this study, which comprises 25-items relating to health-related symptoms common to central sensitisation syndromes. Each question is graded using a 5-point Likert scale anchored 0 (never) to 4 (always), with total possible scores ranging from 0 to 100. Participants who scored ≥40 were deemed to have clinically significant central sensitisation, and indicative of central sensitisation syndrome (CSS) [34].

### Data analysis

Descriptive statistics were used to summarise the characteristics of the participants. All data distributions were explored for normality (visualisation and Shapiro-Wilk normality test) to determine the suitability of parametric or non-parametric statistical tests. Differences in age and IRSD between the participants and those that declined to participate were analysed using the Mann-Whitney *U* test as they were not normally distributed. Gender ratios were compared using the chi-squared test. If a questionnaire (MOXFQ, CES-D, CSI, PCS) was returned with > 50% of the items missing it was excluded from the analysis. Missing items were scored as zero and missing data were not imputed.

Multivariable linear regression was used to determine whether the severity of foot pain as measured by the MOXFQ-index score is associated with various demographic/social (age, sex, BMI) and psychological (depressive symptoms, CSS, pain catastrophising) predictors. A hierarchical multiple linear regression model was employed to establish (a) the variance in MOXFQ-index than can be accounted for by demographic variables (all of which have existing, well-established relationships with musculoskeletal pain), and (b) the extent to which psychological variables explain additional variance in MOXFQ-index score (established relationships in the literature, but less so in this cohort) beyond the influence of demographic variables. Consequently, block 1 comprised age, sex and BMI, and block 2 added depressive symptoms, CSS, and pain catastrophising. Formal collinearity diagnostics were conducted, and standard homoscedasticity and normality checks of residuals were carried out for the final models to ensure validation, and zero-order correlations between predictor variables were also inspected to ensure no problematic collinearity emerged. In all analyses, a *p*-value (two-sided) less than 0.05 was deemed to be statistically significant. All data were analysed with SPSS v27.0 (IBM SPSS Statistics, Armonk, NY, USA).

### Sample size calculation

Given the exploratory nature of this study, we did not specifically calculate a sample size a priori. However, we projected approximately 800 referrals over the 12-month and a 35–40% participation rate giving us approximately 280 participants. With this sample size we would be able to estimate the mean (MOXFQ)-index score with a precision (95% confidence interval) of ±2.8 and estimate the prevalence of the psychological comorbidities of interest with a precision of at least ±0.06. Further, we would be able to detect a predictor that accounts for as little as 3% of the variance in MOXFQ. These calculations were performed using the G*Power software and assuming a standard deviation of 24 in the MOXFQ.

## Results

### Study participants

Total outpatient numbers in public hospitals were lower than anticipated, particularly Category 3 referrals. However, of the 434 patients referred and sent questionnaires, 172 responded, rendering a response rate of 39.6%. The IRSD was not significantly different between the responder and non-responder groups, median (interquartile range (IQR)) 7 (1) versus 7 (2) *p* = 0.253, respectively. The sex distribution was also no different between-groups, with 106 (61.6%) and 159 (60.7%) women in the responder and non-responder groups, χ^*2*^ = 0.039 *p* = 0.844. The responder group, however, was significantly older than the non-responder group, median (IQR) 60.3 (18.3) years versus 48.0 (24.0) years, *p* < 0.001.

Per Table 1, participants had a median (IQR) BMI of 27.6 (7.5) kg/m^2^, with 55 (32%) of the participants being classified as obese (BMI ≥ 30 kg/m^2^). Nearly 75% of participants were taking some form of oral analgesia and 23% of participants reported taking an opioid for their foot complaint. The presence of chronic health conditions was high, with 78.2% of participants reporting one or more. Back pain (56.5%), osteoarthritis (47.9%) and hypertension (33.1%) were the most frequently reported conditions (Table 2). The median (IQR) of the EQ-VAS was 70.0 (30.0) points. A considerable proportion of participants reported moderate problems with mobility (44.7%) and usual activity (38.0%), and over three-quarters (77.8%) indicated either moderate or severe problems with pain or discomfort. Capacity for self-care appeared largely unaffected, with 65.5% of participants reporting no problems, and 42.6% reported no problems with anxiety or depression. Complete data for the five dimensions of the EQ-5D-5L are reported in Table 3.

**Table 1:** Participant characteristics (*n* = 172)

Characteristic	Median (Q1, Q3)^a^
Age, yrs	60.9 (53.4, 71.7)
Sex, *n* female (%)	110 (64.0)
Weight, kg	79.0 (68.0, 93.0)
Height, m	168.0 (160.0, 176.0)
BMI, kg/m^2^	27.6 (24.4, 31.7)
Socio-economic disadvantage (IRSD)	7.0 (6.0, 7.0)
Health-related quality of life (EQ-VAS)	70.0 (50.0, 80.0)
Analgesia, *n* (%)	
Paracetamol	85 (50.3)
Oral NSAIDs	62 (36.9)
Codeine / other opioids	40 (23.7)
Any analgesia, *n* (%)	126 (74.1)
Smoking status, *n* (%)	
Current smoker	10 (5.8)
Ex-smoker	56 (32.6)
Non-smoker	104 (60.5)
Presence of one of more comorbidity (SACQ), *n* (%)	133 (78.2)

^a^Unless otherwise indicated

Data missing from participants (n); height (4), weight (4), IRSD (1), EQ-VAS (3), paracetamol (3), NSAIDs (4), opioids (3), any analgesia (2), smoking status (2)

*Abbreviations: BMI* body mass index, *EQ-VAS* EuroQoL visual analogue scale, *IRSD* index of relative socio-economic disadvantage, *NSAIDs* non-steroidal anti-inflammatory drugs, *SACQ* self-administered comorbidity questionnaire, Q1 = lower quartile, Q3 = upper quartile

**Table 2:** Most frequently reported comorbidities based on the SACQ (*n* = 172)

Comorbidity	***n*** (%)
Heart disease	11 (6.5)
Hypertension	56 (33.1)
Lung disease	10 (5.9)
Diabetes	16 (9.4)
Anaemia or other blood disease	13 (7.7)
Kidney disease	3 (1.8)
Liver disease	2 (1.2)
Depression	29 (17.2)
Cancer	7 (4.1)
Stroke	7 (4.2)
Osteoarthritis	81 (47.9)
Back pain	95 (56.5)
Rheumatoid arthritis	27 (16.8)
Other	33 (19.4)

Values are *n* and percentage of valid responses. Data missing from participants (n); heart disease (4), hypertension (3), lung disease (2), diabetes (2), anaemia or other blood disease (3), kidney disease (2), liver disease (2), depression (3), cancer (2), stroke (4), osteoarthritis (3), back pain (4), rheumatoid arthritis (11), other (2)

Abbreviation: *SACQ* Self-Administered Comorbidity Questionnaire

**Table 3:** Health-related quality of life dimensions, as measured by the EuroQoL-5D-5L (n = 172)

Dimension	Level				
	No problems	Slight problems	Moderate problems	Severe problems	Extreme problems
Mobility	19 (11.2)	36 (21.2)	76 (44.7)	37 (21.8)	2 (1.2)
Self-care	110 (65.5)	35 (20.8)	17 (10.1)	4 (2.4)	1 (0.6)
Usual activity	26 (15.5)	43 (25.6)	64 (38.0)	28 (16.7)	6 (3.6)
Pain / discomfort	2 (1.2)	26 (15.2)	73 (42.7)	60 (35.1)	10 (5.8)
Anxiety / depression	72 (42.6)	49 (29.0)	33 (19.5)	7 (4.1)	7 (4.1)

Values are *n* (%)

Data missing from participants (n); mobility (2), self-care (4), usual activities (4), pain / discomfort (1), anxiety / depression (3)

Abbreviation: *5D-5L* 5-Dimensions-5-Levels

### Pain and disability

Foot/ankle symptoms had been present for a median of over two-years (28.5 months, IQR 46.3 months) prior to referral and bilateral foot pain was reported by 37.2% participants. Multi-site pain was present in 88 (51.2%) participants and 31 (18.8%) participants had no other joint pain throughout their body. Foot pain measured with the MOXFQ was high across the three measured domains and summary score. Median (IQR) scores of 67.9 (35.7) points for walking / standing, 70.0 (25.0) points for pain and 62.5 (37.5) points for social interaction were calculated, with a MOXFQ-index summary score of 65.6 (28.1) points.

### Psychological health

Depressive symptoms were present in 83 (48.3%) of the participants completing the CES-D questionnaire and 66 (38.4%) participants returned scores ≥40 on the CSI, classifying them as having CSS. Of those participants who did complete this questionnaire, nearly one-quarter (24.4%) participants were classified as exhibiting pain catastrophisation on the PCS (Table 4).

**Table 4:** Pain- and psychological health-related measures

Pain Measures	Median (Q1, Q3)^a^
MOXFQ^a^	
Walking / standing	67.9 (50.0, 85.7)
Pain	70.0 (55.0, 80.0)
Social interaction	62.5 (37.5, 75.0)
MOXFQ-index	65.6 (51.6, 79.7)
Depressive symptoms, *n* (%)	83 (48.3)
Central sensitisation syndrome, *n* (%)	66 (38.4)
PCS	
Rumination	6.0 (2.0, 11.0)
Magnification	3.5 (1.0, 6.25)
Helplessness	9.0 (4.0, 15.0)
Pain catastrophisation, *n* (%)	42 (24.4)

^a^Unless otherwise indicated. ^a^In cases of bilateral foot pain, data reported for more painful foot

Data missing from participants (n); MOXFQ-index (5), depressive symptoms (8), central sensitisation syndrome (6), pain catastrophisation (26)

Abbreviations: *MOXFQ* Manchester-Oxford Foot and Ankle Questionnaire, *PCS* Pain Catastrophizing Scale, Q1 = lower quartile, Q3 = upper quartile

### Multiple linear regression analysis

Collectively, age, sex, and BMI accounted for 11.7% of the variance in MOXFQ-index, *F ch.* (3130) = 5.76, *p* < 0.001. BMI was a significant independent predictor of MOXFQ-index: For every 1 kg/m^2^ increase in BMI, MOXFQ-index increased by 1.18 points, *p* < 0.001, with BMI accounting for 10.7% of the unique variance in MOXFQ-index. Age and sex were not significant independent predictors of MOXFQ-index. Shared variance between all predictors accounted for < 1% of the variance in MOXFQ-index.

Beyond the block 1 participant characteristics, the inclusion of pain catastrophising, depressive symptoms and CSS in block 2 accounted for an additional 28.2% of the variance in MOXFQ-index score, *F ch.* (3127) = 19.90, *p* < 0.001. Catastrophising and depressive symptoms were significant independent predictors of MOXFQ-index, with catastrophising explaining 14.4% of the variance in MOXFQ-index score, and depressive symptoms explaining 2.3%. The presence of pain catastrophizing corresponded to an increase in MOXFQ-index of 20.07 points (*p* < 0.001), and depressive symptoms to an increase of 8.97 points (*p* = 0.031), while CSS was not a significant independent predictor of MOXFQ-index in block 2. Shared variance between significant catastrophizing, depressive symptoms and CSS accounted for 11.4% of the variance in MOXFQ-index.

Given the reported *β* values (Table 5), the rankings of predictor importance from highest to lowest are significant catastrophising, BMI, and depressive symptoms; followed by age, CSS and sex. Overall, all six variables combined explained 40.0% of the variance in MOXFQ-index, *F*(6,127) = 14.09, *p* < 0.001. All variance inflation factors were less than 2, with depressive symptoms and CSS yielding the highest scores (1.91 and 1.79, respectively).

**Table 5:** Hierarchical multiple linear regression investigating factors associated with foot/ankle pain severity, as measured via MOXFQ-index

		Unstandardised Bcoefficients (95% CI)	Standardised *β*-coefficients	*P* value
Block 1: Demographic variables	Age	0.17 (−0.09 to 0.43)	0.11	0.201
	Sex (female)	1.25 (−6.10 to 8.60)	0.03	0.738
	Body mass index	1.18 (0.59 to 1.77)	0.33	< 0.001
Block 2: Addition of psychological variables	Age	0.26 (0.04 to 0.48)	0.16	0.021
	Sex (female)	0.59 (−5.67 to 6.84)	0.13	0.853
	Body mass index	0.87 (0.37 to 1.37)	0.24	< 0.001
	Pain catastrophisation (present)	20.07 (12.87 to 27.27)	0.41	< 0.001
	Depressive symptoms (present)	8.97 (0.81 to 17.13)	0.21	0.031
	CSS (present)	1.33 (−6.68 to 9.34)	0.03	0.743

Dichotomously coded variables include sex (males = 0, females = 1), pain catastrophising (1 = catastrophisation, 0 = non-catastrophisation), depressive symptoms (1 = present, 0 = absent), and CSS (1 = present, 0 = absent)

*Abbreviations: CI* confidence interval, *CSS* central sensitisation syndrome, *MOXFQ* Manchester-Oxford Foot and Ankle Questionnaire

## Discussion

This study is the first to explore the prevalence and nature of these specific psychological comorbidities as they relate to foot/ankle pain amongst a public hospital waiting-list cohort. Pain catastrophising, CSS and depressive symptoms were all demonstrated to be prevalent in this group. Our model found 40% of the variance in a patients’ foot/ankle pain can be explained prior to specialist evaluation and without a formal orthopaedic diagnosis.

Pain catastrophising had the most significant association with foot/ankle pain severity in our regression model. Catastrophising is a set of maladaptive beliefs which magnify the threat value of pain stimuli and the attention paid to these as well as the tendency to feel helpless to reduce the response in the face of repeated painful exposures [33, 35]. Research has demonstrated consistent associations between measures of catastrophising and pain-related clinical outcomes including severity, occupational-functional recovery and response to treatment which are maintained even when controlling for co-morbid depression [36–39]. The precise nature of factors that predispose to pain catastrophising are not yet clearly understood and are likely multifactorial, however it has been reported that pain frequency plays an influential role in moderating the effect of catastrophising on symptoms [40]. This is of interest, because-in-a-cohort-of-patients-referred-to-foot-and-ankle specialist clinics almost all have frequent pain experiences which meaningfully affect their quality of life. Understanding, measuring where possible, and-intervening-in-pain-catastrophisation-should-therefore-be of interest to orthopaedic specialists, however research suggests that surgeons are not able to routinely identify the presence of pain catastrophising in their patients during initial consultation [41]. It is likely this is the case in the Australian public health system where patient numbers are high, resources-are-finite-and-consultations-may-be-brief-as-a-result. These results clearly suggest that pain catastrophising is related to reported symptom severity in patients referred with foot-and-ankle-pain.--Although the directionality of the association between the foot/ankle pain and catastrophising association warrants further investigation, catastrophising could provide a useful -therapeutic target for non-operative and pre-operative interventions. Further, research is required to identify effective interventions catastrophising and their impact on patients’ foot/ankle pain.

The positive association of pain severity with BMI in this group is in keeping with a previous study investigating foot/ankle pain in patients on non-urgent waiting-lists [42]. This present study, however, found that the association persists even after adjusting for psychological health conditions which strengthens the already established, independent association between BMI and pain. The association of BMI with chronic musculoskeletal foot pain has been well described [43], and although the presumption has historically been that this is primarily related to mechanical loading (which does likely play a role) the non-mechanical relationship between increased adiposity and pain should not be discounted [44]. Elevated BMI may be related to pain via mechanisms related to chronic low-grade inflammation, comorbid psychological distress and even impaired sleep. Weight loss via bariatric surgery has been associated with a reduction in foot/ankle pain [45], although whether other forms of weight loss have similar effects on pain is yet to be investigated, but could be helpful for this group given the prolonged waiting times for appointments and may present an opportunity for such interventions to be applied.

Depressive symptoms as measured by the CES-D were highly prevalent (48.3%) and were significantly and independently associated with of pain severity (though with less strength than pain catastrophising) in our regression model. The concordance with previous research is also striking, other studies have found similarly high prevalence rates, between 21 and 30%, and that depressive symptoms are 2–4 times more likely to be present in conjunction with moderate to severe foot pain, as well as linearly related to symptom severity when controlling for other patient specific factors [46–48]. Clinical depression has a well described bidirectional relationship to pain [49, 50] and depressive symptoms have been associated with both higher patient expectations [48, 51] and worse patient reported outcomes following arthroscopic surgery [52], lower limb arthroplasty [38, 53–55] and foot and ankle surgery [19, 56]. The direction of effect with respect to depressive symptoms in this cohort cannot be inferred due to study design, though treatment of depression pre-operatively has been shown to not meaningfully affect the outcome of surgery in arthroplasty patients in previous research [57].

The findings are in keeping with the literature describing the complex reciprocal interplay of physiological and psychosocial factors influencing the presentation of musculoskeletal pain. Taken in context of the available literature, the results of this study suggest that a meaningful proportion of foot and ankle patients are at risk of poorer outcomes related to their mental health and BMI, and that managing their expectations of surgery in this context may be warranted. Directions for further research could include investigation of how BMI and psychological health measures change over time and what influence early pre-operative assessment and/or management strategies may have on treatment outcome.

Limitations of this study include its cross-sectional design and inherent difficulties with drawing any casual relationships between variables. Indeed, an organic foot complaint that becomes chronic in nature could feasibly provoke a decline in psychological health, while poor psychological health could reduce the threshold for pain perception. Additionally, specific foot/ankle diagnoses were not known and thus not included in the model. These could be important contributors to the psychology-pain relationship and would be a target for inclusion in future studies. This study was also conducted by mail and relied on self-reported information and rendered a response rate of 40%. Older patients were demonstrably more likely to respond to the written questionnaires. It is possible that younger patients who are present on waiting-lists are less inclined to communicate by mail compared to online means due to their communication habits and preferences. As such, the responders may not reflect whole spectrum of those on waiting-lists. A higher response rate may have been yielded by including reminder letters to non-responders. Despite this, our response rate and responder characteristics are consistent with those obtained by similar mail surveys of this group [42]. Finally, it should be noted that, whilst significant, our model could not account for 60% of the variance in patients’ foot/ankle pain. Future models could incorporate variables such as clinical diagnosis, multimorbidity and polypharmacy, as well as additional psychological variables (e.g., anxiety).

This study has several strengths. Firstly, it provides a previously unrecognised insight into the psychological distress patients entering public foot/ankle orthopaedic waiting-lists are under. Secondly, it uses validated measures of psychological health and foot/ankle pain. Furthermore, despite the response rate of 39.6% and proportionately lower response rate in younger patients, the cohort studied is comparable to those from previous studies of foot and ankle clinic waitlists in the public sector which constitutes a very large proportion of the total pool of patients seeking care [10, 11, 58]. Finally, this study provides further evidence of the complex, additive effect psychological comorbidities and obesity play in a person with chronic pain.

## Conclusion

In conclusion, this study has given new insight into the prevalence and nature of specific and measurable psychological vulnerabilities in a cohort of foot and ankle patients on a public hospital waiting list. The results demonstrate that psychological comorbidity is common and that pain catastrophising, BMI and depressive symptoms continue to play an important role in pain perception. Interestingly, this study has found that 40% of foot/ankle pain can be explained by factors that orthopaedic surgery does not treat directly. Identifying people with pain catastrophisation, elevated BMI and depressive symptoms may be useful if they could be potential targets for inclusion in tailored non-operative or pre-operative management strategies to reduce pain and improve function.

## Data Availability

The data that support the findings of this study are available from the corresponding author upon reasonable request.

## Abbreviations

5D-5L: 5-Dimensions-5-Levels
BMI: Body mass index
CES-D: Center for Epidemiological Studies-Depression
CI: Confidence interval
cm: Centimetre
CSI: Central Sensitization Inventory
CSS: Central sensitisation syndrome
EQ: EuroQoL
HRQoL: Health-related quality of life
IBM: International Business Machines
IQR: Interquartile range
IRSD: Index of Relative Socio-economic Disadvantage
MOXFQ: Manchester-Oxford Foot and Ankle Questionnaire
NSAIDs: Non-steroidal anti-inflammatory drugs
NY: New York
PCS: Pain Catastrophizing Scale
SACQ: Self-Administered Comorbidity Questionnaire
SED: Socio-economic disadvantage
SF-36: 36-Item Short Form Survey Instrument
SPSS: Statistical Package for the Social Sciences
USA: United States of America
VAS: Visual analogue scale
